# Efficiency of Melatonin as a Sedative for Auditory Brainstem Response in Children

**DOI:** 10.3390/audiolres10020009

**Published:** 2020-11-14

**Authors:** Anass Chaouki, Zineb El Krimi, Amine Mkhatri, Oukessou Youssef, Sami Rouadi, Reda Abada, Mohamed Roubal, Mohamed Mahtar

**Affiliations:** Faculty of Medicine of Casablanca, Casablanca 20 000, Morocco; anasschaouki.ac@gmail.com (A.C.); zineuub@gmail.com (Z.E.K.); oukessouyoussef@gmail.com (O.Y.); samisami@gmail.com (S.R.); Redabada@gmail.com (R.A.); roubarouba@gmail.com (M.R.); mahtarino@gmail.com (M.M.)

**Keywords:** auditory brainstem response, melatonin, pediatric audiometry, hearing loss, sedation

## Abstract

*Introduction*—Although auditory brainstem response (ABR) testing is among the most frequently used investigations in pediatric audiology and it often requires sedation or general anesthesia. In recent years, melatonin has been successfully used as an alternative way of inducing sleep, particularly in children undergoing magnetic resonance imaging (MRI) or electroencephalography (EEG). *Purpose*—To assess the effectiveness of orally administered melatonin as an alternative to sedation or general anesthesia during ABR testing. *Method*—In total, 33 children with suspected hearing loss underwent ABR tests in melatonin-induced sleep. Each patient received an initial dose of 5 mg, which was re-administered in case of failure to obtain sleep. Click-induced ABR tests were performed on both ears. *Results*—ABR tests were successfully performed in 72.7% of the patients. The average total length of time needed to obtain sleep and complete the ABR testing was 45 min. There was no significant difference between the patients who completed the examination and those who did not in terms of age or psychomotor development. There was a statistically significant association between receiving a maintenance dose and successful completion of the test (*p* < 0.001). There was also a significant connection between the degree of hearing loss and the success rate of the ABR tests (*p* < 0.001). *Conclusions*—Melatonin-induced sleep is a good and safer alternative to anesthesia to perform ABR testing in young children. It is easily administered, tolerated by the patients, and accepted by parents.

## 1. Introduction

The evaluation of hearing loss through subjective methods is often difficult and sometimes impossible in children. Therefore, objective means are currently employed to assess hearing in children and determine the hearing threshold.

The auditory brainstem response (ABR) is the most widely used objective method to test the auditory pathway. Though, when used on a child, it often requires sedation [1,2]. A multitude of protocols have been used to achieve sedation but they all expose the subjects to numerous adverse effects [3,4].

Melatonin is a natural hormone that regulates the circadian rhythm. When administered orally, it can induce sleep, without exposing children to the risk of upper airway obstruction, and, thus, does not needing close monitoring. Additionally, there have been controlled studies about the long-term use of melatonin that have not shown any side effects [5,6]. Therefore, it has been used as an alternative to sedation in children undergoing magnetic resonance imaging (MRI) scans or electroencephalography (EEG) [7,8].

The aim of this study was to assess the effectiveness of melatonin as an alternative to anesthesia in ABR tests and seek any factors which influence its efficiency.

## 2. Patients and Methods

In total, 33 children with suspected hearing impairment were included in this study, which was conducted at the Department of Otorhinolaryngology of the hospital of 20 August 1953. Each patient underwent ABR tests during melatonin-induced sleep. We used synthetic melatonin in the form of drops, administered orally. Written consent was obtained from the parents beforehand. Each patient, regardless of their age, received an initial dose of 5 mg, equivalent to 12 drops. In case of a failure to obtain sleep, an additional 5 mg was added after 30 min.

The electrodes were placed after the child had fallen asleep. We performed click-evoked ABR tests at 500, 1000, 2000, and 4000 Hz. ABR testing was performed on the right side first, and then on the left side. We started with a noise level of 70 dB. If necessary, the volume was increased up to 100 dB or brought down to a minimum of 20 dB. The investigation was terminated once the ABR test was completed, or if the child woke up or did not fall asleep.

We classified sleep as “good” when the children slept for the duration of the examination, “brief” when the children slept initially and then woke up but did not move during the rest of the test, or “absent” when the children had never closed their eyes, or when sleep was intermittent with regular movements, preventing the recording.

## 3. Results

In total, 66 ABR tests were carried out under melatonin (66 ears—33 children). Their ages varied from 5 months to 4 years, with an average of 2 years and 8 months. Out of our patient population, 17 had a medical history relevant to their condition (Table 1). The other 16 patients had no notable medical history.

### 3.1. Percentage of ABR Tests Completed through the Use of the Melatonin

ABR tests were completed on both ears in 72.7% of the patients (24 children). A failure to complete the ABR test was noted in nine patients (27.3% of the patients). We noted that five of these children were anxious, one woke up at the beginning of the ABR test, and three woke up while placing the electrodes. Therefore, ABRs could not be recorded.

A subgroup calculation showed that there was no significant difference between the age of the patients whose ABR tests were successfully completed and the age of the patients with failed ABR tests (*p* = 0.23).

In a similar fashion, there was no significant difference concerning the success of the ABR tests between children exhibiting a psychomotor delay and children with a normal psychomotor development (*p* = 0.19).

### 3.2. ABR Tests Duration

The melatonin took between 15 and 55 min to take effect, with an average of 30.39 min. The duration of the ABR tests varied from 10 to 30 min, with a mean duration of 15 min. The average total length of time necessary to complete the examination, starting from achieving sleep, to placing the electrodes and completing the ABR testing, was 45 min. Out of 33 patients, 16 required a maintenance dose (48.5% of the children).

In our study, there was a statistically significant association between receiving a maintenance dose and successfully completing the ABR tests (*p* < 0.001).

### 3.3. Percentage of Children Who Reached Sleep on Melatonin

Twenty two patients had a “good” sleep (66.6%); two children had a “brief” sleep (6.1%); nine children did not sleep (27.3%).

### 3.4. Hearing Threshold and Success of the ABR Tests

The presence of an ABR response was determined by the existence of a wave V. As such, a response was positively recorded in 35 ears (18 left ears and 17 right ears). Wave V was absent in 13 ears (six left ears and 7 right ears), excluding the patients who failed to complete their ABR tests. The degree of hearing loss in each ear is represented in Table 2.

There was a significant association between the success rate of the ABR tests and the degree of hearing loss (*p* < 0.001).

## 4. Discussion

The use of ABR testing under sedation or general anesthesia is currently the gold standard in pediatric audiometry. In recent years, there have only been a few studies seeking to establish the efficiency of melatonin as a sleep-inducer and replacement of anesthesia in ABR testing.

Our study showed that ABR testing during melatonin-induced sleep was successful in 72.7% of the patients. This success rate was relatively close to what is found in other studies. Guerlain [9] carried out a study comparing melatonin to pentobarbital as a sleep-inducer for children undergoing ABR testing. In the melatonin group, 77% of ABR tests were done successfully. Schmidt [10] reported a successful completion of ABR tests in 86.7% of their patients, while Casteil [11] found a success rate of 90% in their study.

The length of time necessary the complete ABR testing in our study was 45 min, which is consistent to what is found in literature, regardless of the method used to achieve sedation or sleep [9,10,11,12].

While both Casteil and Guerlain have not found the degree of hearing loss to be a significant factor influencing the course of the examination, our study proved otherwise. Casteil found that the existence of a psychomotor delay could affect the efficacy of melatonin. This does not agree with our results. The age of the patients was not found to be of significant importance in any study.

When comparing melatonin to pentobarbital, Guerlain did not find a significant difference between the two groups in terms of success rate and hearing thresholds. Sleep duration was, however, significantly longer in the group receiving pentobarbital.

No side effect to the use of melatonin has been reported in literature, even in children with a neurological condition [13]. On the other hand, side effects have been described with the use of anesthesia or even pentobarbital [14,15,16,17,18]. Consequently, melatonin seems like a safer method to induce sleep and achieve ABR testing.

## 5. Conclusions

In conclusion, melatonin is a good and efficient way of conducting ABR tests in children with suspected hearing loss. It offers a risk-free alternative to anesthesia, is widely accepted by parents, easy to use, and does not necessitate monitoring, allowing for an earlier diagnosis and subsequent treatment of hearing loss in young children.

## Figures and Tables

**Table 1:** Table showcasing the medical history of the patients undergoing auditory brainstem response (ABR) testing.

Medical History	Number of Patients
Familial hearing impairment	4
Meningitis	3
Autism	2
Perinatal asphyxia	2
Cardiac malformation (IVC)	1
Exposition to loud noises during infancy	1
Neonatal jaundice	1
Bilateral otitis media (treated with ear tubes)	1
Psychomotor development retardation	1
Down syndrome	1
No notable medical history	16

**Table 2:** Degree of hearing loss of each ear, as recorded in the ABR tests.

	Normal Hearing	Mild Hearing Loss	Moderate Hearing Loss	Severe Hearing Loss	Profound Hearing Loss
*Right Ear*	9	3	4		8
*Left Ear*	8	5	3	1	7
*Total*	17	8	7	1	15
